# Synergistic Therapeutic Effects of Chitosan and Royal Jelly

**DOI:** 10.3390/polym17212872

**Published:** 2025-10-28

**Authors:** Raluca-Marieta Toma, Adriana Băran

**Affiliations:** Ilie Murgulescu-Institute of Physical Chemistry, Romanian Academy, 202 Splaiul Independenței, 060021 Bucharest, Romania

**Keywords:** chitosan, royal jelly, synergistic mechanism, targeted delivery

## Abstract

The fields of healthcare and pharmaceutical science are increasingly focused on developing innovative and effective treatments. This trend is driven by a growing consumer demand for natural, sustainable, and highly functional polymer-based products. This study focuses on two biomaterials: chitosan and royal jelly. Chitosan, a linear polysaccharide derived from chitin, is well-regarded for its hemostatic and antimicrobial properties, making it an excellent candidate for wound healing applications. Royal jelly, a nutrient-rich secretion from honeybees, represents a complex mixture of proteins, lipids, vitamins, and antioxidants, notably 10-hydroxy-2-decenoic acid (10-H2DA). It is known for its anti-inflammatory, antioxidant, and regenerative effects on the skin. While the individual benefits of chitosan and royal jelly are well-documented, there is a significant research gap concerning their synergistic application in various treatments such as topical formulations, wound healing, regenerative medicine, and delivery transport processes. Ultimately, this review concludes that the synergistic effects of chitosan and royal jelly could provide a material platform with a superior dual-action profile, integrating the structural and antimicrobial benefits of chitosan with the powerful regenerative and anti-inflammatory effects of royal jelly. This synergy strongly supports their utility in developing next-generation, high-performance natural bioproducts for wound healing, bone regeneration, agriculture, or aquaculture applications.

## 1. Introduction

Current healthcare research emphasizes using functionalized biopolymers as matrices for targeted drug delivery [1]. The success of this approach is critically dependent on developing a comprehensive understanding of how the active compounds permeate biological tissues [2]. Due to their inherent versatility, biocompatibility, and biodegradability [3], biopolymers have emerged as key players in this field [3]. These natural materials are easily processed and shaped into diverse structures, including edible films [4,5], hydrogels [6], microspheres, fibrils, and nanocomposites, making them extensively applicable in related areas such as medicine, pharmacy, cosmetics, agriculture, or ecology [7,8,9,10,11].

One representative biopolymer is chitosan (CS), which is primarily found in the exoskeletons of aquatic organisms. It possesses a fully responsive structural backbone which consists of functional amino (-NH_2_) groups [12]. Because of its abundance in nature and interesting structural architecture, CS is known to be a promising ingredient considered to be very useful in helping and improving scalp skin and hair follicles [13], but there has been limited exploration of it in the niche of applied research, especially in topical formulations [14]. With its main contributions in rejuvenation and regeneration processes [15], CS is still shaping dreams across the scientific barrier, due to its bioactive properties and non-toxicity [16]. It has several molecular weights on the market (low, medium, and high molecular weight) and its moisture retention capacity depends on this [17]. The literature shows that additional chemical modification to its backbone improves moisture adsorption processes better than hyaluronic acid products [13], exploiting the cationic character of CS, which conduct to its adsorption on the negatively charged skin surface [18]. It can work by forming a protective film to maintain a moist wound environment, promoting cell adhesion and fibroblast proliferation, and inhibiting microbial growth [19]. CS also serves as a versatile drug-delivery vehicle [20], improving the solubility and delivery of other active pharmaceutical agents for treating various skin conditions [21].

Royal jelly represents another attractive candidate, a creamy yellowish powder secreted by young worker bees for feeding the queen and other young compatriots. It is totally synthesized by the bees in the hypopharyngeal and mandibular glands and it has a rich content of nutrients such as proteins, vitamins, sugars, and lipids [22]. Moreover, royal jelly is strongly antibacterial, which makes it an ideal ingredient in personal care products [23]. Its proliferative effect was highlighted in a recent article, which described the characterization and evaluation of a soft gel–cream for topical use, based on studies conducted on fibroblast cells [24]. In addition to this, for many years, royal jelly has been subjected to investigations on its pharmacological properties in the healthcare domain due to its anticancer and protective effects on tumor growth [25].

Over the past few years, there has been an upward trend focusing on studying and performing complex investigations regarding the combined properties of CS and royal jelly for obtaining bio-enhanced materials, as follows: loaded nanoparticles [26]; nanofibers produced for wound dressing [27]; scaffolds used in regenerative medicine [28]; green synthesis of composite films [29]; hydrogel delivery systems [30]; packaging [31]; and cosmetic formulations [24]. Our main purpose is to clarify and to bring to the forefront the latest research in the field, exploring the synergy between CS and royal jelly with potential medical applications. Thus, we aim to elucidate how CS can act as a delivery vehicle for royal jelly’s active compounds, thereby improving their stability and effectiveness. The goal of this research is to advance the field of sustainable therapeutics by demonstrating the potential of this powerful natural synergy for applications such as advanced wound care, drug delivery, and skin rejuvenation.

## 2. Chitosan in Therapeutics

### 2.1. Structure and Key Properties

**Figure 1:**
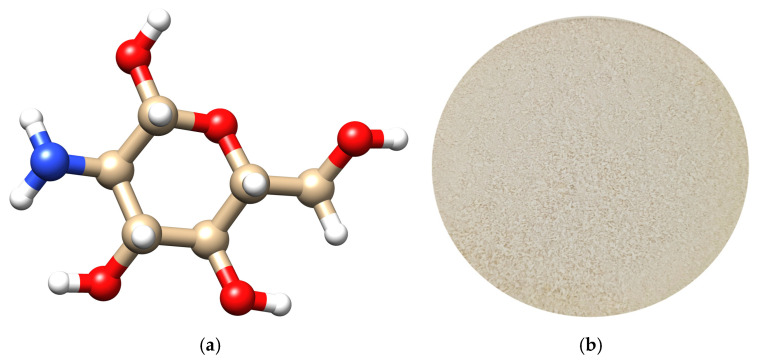
(**a**) Representation of a D-glucosamine unit using Avogadro software (version 1.102). The color code is assigned as follows: red—oxygen; blue—nitrogen; beige—carbon; and white—hydrogen; (**b**) CS powder.

For several decades, naturally derived materials have captivated the scientific community with their unique and complex properties [32]. CS is one of many polysaccharides, widely used in medicine fields, obtained through enzymatic processes from chitin [33]. The backbone is constituted by key components such as D-glucosamine units (Figure 1) linked together through glycosidic bonds (1→4), responsible for structural modifications in different environments, and further essential for designing materials with tailored properties [33]. Native CS requires acidic conditions (acetic acid, lactic acid) for positively charging its amino groups [11], allowing the formation of strong electrostatic bonds with negatively charged molecules, such as nucleic acids, proteins [34], or even small crosslinkers such as linear (tripolyphosphate) and circular polyphosphates (phytic acid) [35,36,37]. The ability to self-assemble makes it ideal for designing hydrogels, films, nanoparticles, and other functional materials for drug delivery, tissue engineering, and wound healing [38].

At neutral or alkaline pH values, the amino groups are deprotonated, and the chains have a tendency to coil up, leading to lower viscosity and eventual precipitation [39]. The viscosity parameter is critical as it dictates the material’s properties for various applications. Specifically, a higher molecular weight (>300 kDa) leads to longer polymer chains in solution which become entangled, restricting their movement and thereby significantly increasing the overall viscosity [40], which is suitable for developing scaffolds, thick gels, and tissue engineering applications [41]. Low-molecular-weight (<100 kDa) solutions are ideal for advanced formulations such as nano- and microparticles used in controlled drug delivery [42]. Medium-molecular-weight (190–310 kDa) CT exhibits antibacterial and antiviral activity, making it suitable for functional coatings, wound dressings, and development of thick gels [42]. The DDA (deacetylation degree) refers to the presence of a number of D-glucosamine units in the polymer chains. A higher DDA value means more free functional amino groups (−NH_2_), arranged for easy protonation in acidic media, leading to strong electrostatic repulsion between chains, which causes them to expand and contribute to an increase in viscosity [39]. In acidic conditions (lower pH values), amino groups become protonated and influence the polymer chains to repel each other, uncoil, and stretch out, conducting to them being so-called “smart” or “stimuli-responsive” biopolymers [43]. This “switch-like” behavior is what makes CS so valuable for applications like controlled drug delivery, tissue engineering, or scaffolds, where it can form a stable matrix, with this being the most defining bioproperty of CS, next to antimicrobial, biocompatible, and biodegrading functions [44].

### 2.2. Role of CS in Wound Healing Treatments

A strong relationship exists between natural polymers and wound healing therapy, with a particular focus on the role of CS in active dressings, which promotes cellular proliferation and protects against infections [45]. Over the years, CS has emerged as a pioneer in wound healing treatments, working as a highly effective hemostatic agent [46]. The body’s immediate response to a skin injury is bleeding, which occurs through a vasodilatation process, in which blood vessels expand to allow blood plasma and immune cells to reach the site of the wound [47]. In the first stage, CS can be efficiently used in erythrocyte aggregation and can inhibit fibrinolysis [19]. Positively charged amino groups are strongly attracted to the negatively charged cell membranes of red blood cells and platelets. This interaction causes these blood components to rapidly aggregate, forming a clot, which effectively seals the wound and stops the bleeding [48]. Following this, platelet aggregation and clot formation begin, which precedes the inflammatory phase [49]. In this phase, CS can assist in bacterial fighting through cell wall disruption, or can interact with microbial DNA or chelate metal ions [49]. The proliferative phase then overlaps with the end of inflammation and is characterized by the formation of new blood vessels, a process known as angiogenesis [50]. After a wound is sealed, the inflammatory response begins, as well as the risk of bacterial infections. The positive charge of CS allows it to disrupt the negatively charged outer membranes of possible bacteria and fungi, leading to the death of the cells. This helps prevent the wound from becoming infected, reducing inflammation and creating a clean area for healing [50]. Finally, the remodeling phase completes the healing process with the formation of scar tissue. This scarring results from an imbalance where the synthesis of collagen surpasses its degradation [51]. As the new tissue is formed and matures, CS scaffolds are gradually broken down by enzymes in the body, leaving no residue behind. This allows the newly formed tissue to fully integrate and strengthen naturally. Here, CS plays a critical role, influencing specific cells involved in these wound healing stages. Table 1 below presents various CS-based materials and their respective applications in wound dressings [52].

### 2.3. Essentials in Drug Delivery

**Figure 2:**
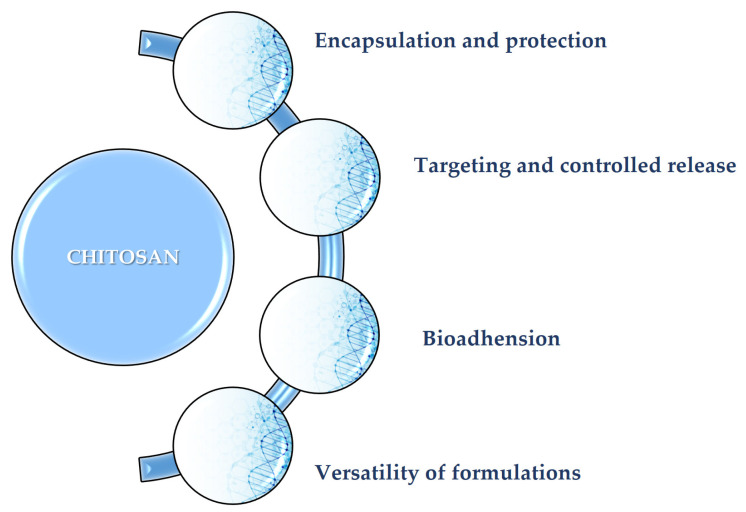
Key roles of CS in advanced drug delivery systems. This figure was designed by using SmartArt graphic creation powered by PowerPoint Tools.

The primary role of CS as a protective carrier for various therapeutic agents (small hydrophobic molecules), delivering them to a specific site in a controlled manner, leads to more efficient, more targeted, and safer therapeutic outcomes (Figure 2) [66]. Each formulation is suited for a different drug and route of administration, whether it is an oral drug, an injectable hydrogel, or even a topical product [67]. Many drugs can be exposed and degraded by the enzymes in the body, before they reach their final targets [67]. The cationic profile of CS facilitates electrostatic binding to these molecules, enabling the creation of smart materials (e.g., protective nano-/micro-particles, gels, and fibers). These structures are vital for the safe encapsulation and transport of therapeutic agents [68]. The release of the drug from the CS shell is typically pH-dependent [69]. The pH value is a crucial parameter in drug transportation. For instance, a drug-loaded chitosan (CS) nanogel can remain stable in the bloodstream, which has a neutral pH, but will then swell or degrade in the more acidic environment of a tumor or inflamed tissue. This allows the therapeutic agent to be released precisely where it is needed [70]. Such controlled release mechanisms are vital for minimizing systemic side effects and significantly improving the effectiveness of the encapsulated molecule. CS has a strong adhesive property to mucosal tissues (e.g., inside the nose, mouth, or intestines) [71,72]. This bioadhesion process is possible due to positive charging of CS, which interacts with the negative charges on cell surfaces [73]. This critical property allows CS-based delivery systems to stick firmly to the site of action, consequently increasing drug absorption and improving the drug’s overall bioavailability [74].

### 2.4. Role of Chitosan in Cosmetics and Applied Dermatology

CS excels as a moisturizing and soothing agent, but research in the niche of cosmetics fields is still ongoing and limited [75]. CS can retain a significant amount of water and form a thin, breathable film on the surface of the skin [76] that acts as a barrier, effectively reducing trans-epidermal water loss and helping to maintain hydration and elasticity at the level of the skin layers [76]. This fact is highly valued in the formulation of anti-aging creams, lotions, and masks, where long-lasting hydration is a key long-term benefit [77]. The antimicrobial properties of CS, previously discussed in the context of wound healing, are also relevant in cosmetics and dermatology. It can be incorporated into products designed for acne-prone skin to help control bacterial proliferation [78]. Additionally, CS has a soothing effect on irritated skin, making it useful in formulations for post-cosmetic procedures or for alleviating minor skin irritations [79]. Similar to its role in drug delivery, CS can act as a sophisticated delivery system for active ingredients in cosmetic products [80]. It can encapsulate a diverse array of compounds, such as vitamins, antioxidants, or plant extracts, protecting them from degradation and ensuring their controlled release into the upper layers of the skin [80,81]. This enhances the efficacy of the active ingredients and allows them to work where they are most needed. In summary, CS is a multifunctional ingredient in cosmetics and dermatology, offering both fundamental benefits like hydration and protection and advanced functionalities like targeted delivery of active compounds.

## 3. Royal Jelly: Composition and Therapeutic Potential

### 3.1. Origin and Composition of Royal Jelly

**Figure 3:**
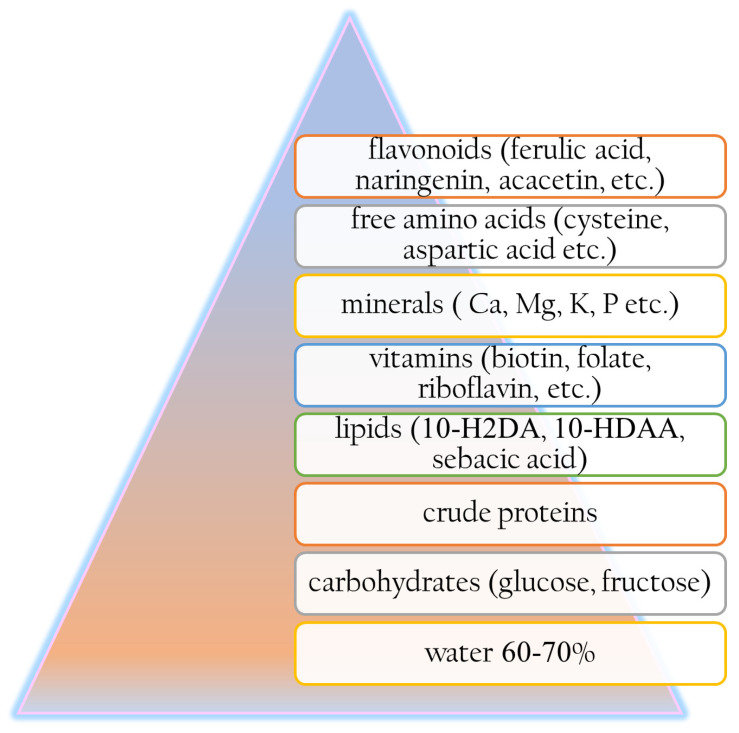
The composition of royal jelly.

The marvelous world of active and nourishing ingredients such as bee derivatives used for skin routines can be traced back to ancient times. Over the last thousands of years, bee derivatives including royal jelly have been used for various skin conditions, from wound healing to ulcers of the lips or baldness treatments [82]. The earliest recorded medical remedy dates from the Sumer period and included the external use only of a topical product based on a mixture of honey, oil, spread river dust, and water [83]. In ancient Egypt, royal jelly powder was used even by Queen Cleopatra and considered to be her personal secret moisturizing ingredient [84]. In the dynasties of ancient Egypt, royal jelly achieved peak recognition and became a symbol of the strength and majesty of the pharaohs, who knew the benefits of its consumption [85]. Later in history, Aristotle was the first person who discovered its role in bee society, associating its consumption with intellectual capacity improvements [85]. The term “Royal Jelly” was first coined in the 18th century by the French scientist René Antoine de Réaumur to name the specific feed designated for the queen bee [85]. In the last few decades, royal jelly has achieved fame due to its whitening, tonic, moisturizing, anti-aging, cell rejuvenation, hydrating, healing, and soothing properties [86]. Royal jelly powder has a pH between 3.6 and 4.2; it is slightly soluble in aqueous media and contains a high percentage of water, between 60 and 70% (Figure 3) [23]. Its solubility depends on the specific composition of the sample and environmental conditions (pH, temperature). Royal jelly’s characteristic solubility profile in common solvents is outlined below (Table 2).

**Figure 4:**
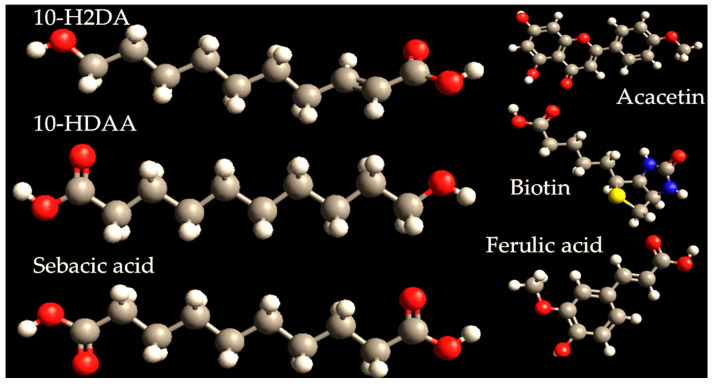
Representative chemical structures of sebacic acid, 10-H2DA, 10-HDAA, acacetin, ferulic acid, and vitamin B7 (biotin), designed by using Avogadro software. The color code is assigned as follows: red—oxygen; blue—nitrogen; grey—carbon; white—hydrogen and yellow—sulfur.

Royal Jelly’s composition can vary in relation to harvest, region, seasons, or the metabolism of nurse bees [87]. Based on its unique composition, royal jelly is primarily characterized by the Major Royal Jelly Proteins (MRJP family, 1–9) (summarized in Table 3) and a rich lipid profile (shown in Figure 4). This profile includes key fatty acids like 10-hydroxy-2-decenoic acid, 10-hydroxydecanoic acid, and sebacic acid, alongside significant amounts of flavonoids, vitamins, sugars, free amino acids, and trace minerals [22]. More than 45% is represented by MRJP1, also known as apalbumin 1 or royalactin, known as a queen marker, the most studied protein [88]. The MRJP family contains high amounts of amino acids such as arginine, histidine, (iso)leucine, lysine, phenylalanine, or tryptophan [89]. Leveraging its rich biological composition, royal jelly is now successfully incorporated into a wide range of advanced delivery vehicles, including topical ointments, scaffolds, nanofibers, nanoparticles, composite films, and gels [90].

**Table 2:** Solubility profile of royal jelly.

Solvent	Solubility and Additional Notes
aqueous solution	slightly soluble [91]
saline solution, NaCl 0.9%	slightly soluble [92]
ethanol	large amounts of lipids and proteins are soluble [93]
acetonitrile	the most suitable solvent for the extraction of fatty acids (10-HDA and 10-H2DA acids) [90]
chloroform/methanol	fatty acid extraction [90]

**Table 3:** Protein screening and their main contributions.

Protein	Contributions	References
MRJP1	immune modulator	[88,94]
MRJP2	proliferative effect	[95]
MRJP3	anti-inflammatory properties and protein effector	[96]
MRJP4	nutritional function	[97]
MRJP5	high protein content	[98]
MRJP6	anti-inflammatory, immunomodulatory, antimicrobial, anticancer, and antihypertensive properties	[99]
MRJP7	antimicrobial, anti-inflammatory, and antioxidant properties	[99]
MRJP8	broad-spectrum antimicrobial activity	[100]
MRJP9	antibacterial and antifungal activities	[100]

### 3.2. Therapeutic Effects of Royal Jelly

**Figure 5:**
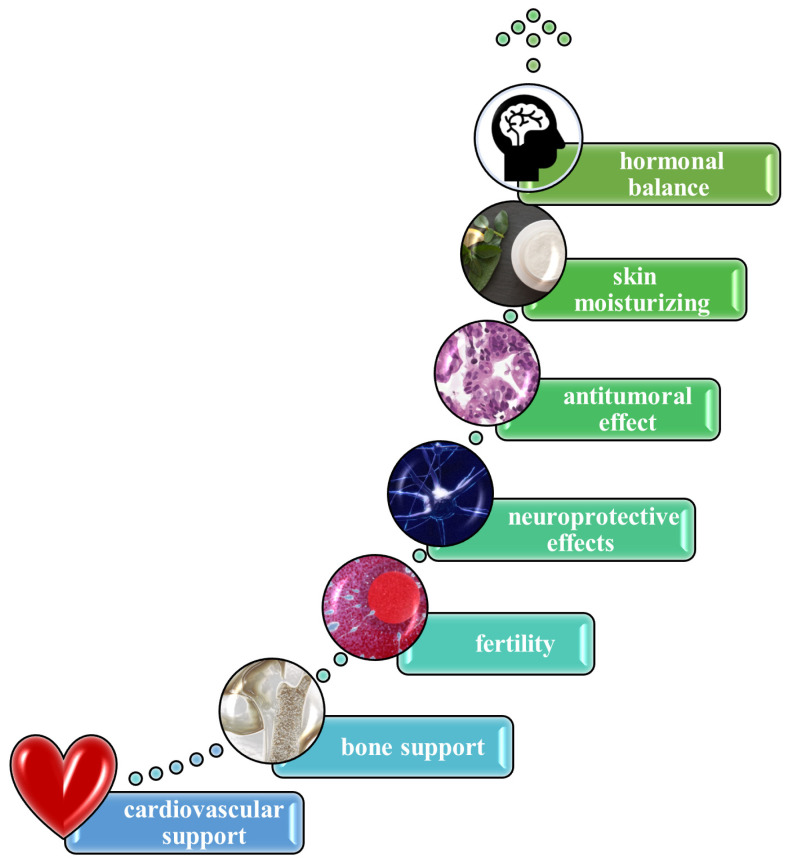
A few of the benefits of royal jelly. Stock images were generated by using SmartArt graphic creation powered by PowerPoint Tools.

Royal jelly exhibits a broad spectrum of therapeutic effects by acting as an anti-inflammatory, antioxidant, antimicrobial, and immunomodulatory agent, supported by its complex composition of proteins, peptides, fatty acids, vitamins, and other bioactive compounds (Figure 5) [86]. Preclinical and clinical studies suggest royal jelly may improve immune function [101], support skin health [102], regulate blood sugar [103] and cholesterol [104], and show promise in enhancing cognitive function [105] and fertility [106]. Specific compounds like 10-HDAA and royalactin are linked to neuroprotective, cardio-protective, and hormonal balance effects [107]. Royal jelly’s interaction with apolipoproteins primarily concerns its potential to modulate lipid metabolism and cardiovascular health [108]. Royal jelly can also contribute to enhancing collagen production [109]. It also contains vitamins that keep the skin hydrated and rejuvenated, preventing the formation of wrinkles and reducing sagginess [110]. Furthermore, these nutrients can help blood circulation at the level of the scalp [111]. Moreover, it can regulate skin moisturizing, depigmenting, photoaging, and whitening by inhibition of the enzyme tyrosinase [112]. In the cosmetic sector, informed consumers know about the remarkable properties of royal jelly, making it a very popular and requested ingredient. With exponential growth on the internet and social media, the royal jelly market is going to reach a valuation of USD 1667.23 million in 2025 [113]. The Asia–Pacific region represents the largest market for royal jelly incorporated in cosmetics and dermatological products, mostly in those used for skin refreshing, regeneration, or rejuvenation [114].

“The royalty” in topical products is very recognized for its use in its fresh form, mixed with other active ingredients, or freeze-dried [115]. In its raw state it can also be included directly in many food and dietary supplements, as well as pharmaceutical products or cosmetics. It is also used in formulations or ointments for healing burns and wounds, usually in small dosages only (from 0.05 to 1%) [116]. A recent study proposed a film-forming system as a compelling option for incorporation of royal jelly, because the formed pellicle can lose volatile components after application to the skin, creating a thin film, which can increase hydration, improving the delivery of the bee derivative through the layers of the skin [117]. It could also act as an epigenetic modulator due to its rich content of polyphenols [118]. Both lifestyle and environmental factors have a huge impact on the skin cells’ memory. Royalactin can work as a potent activator of a pluripotency gene network through modulation of chromatin accessibility, being responsible for the epigenome of queen bees [119]. Due to its zinc, choline, and phenolic content, royal jelly provides antioxidant and neuroprotective properties for further prevention of neurodegenerative diseases [120]. Its proteins inhibit cholesterol absorption at the level of the intestines and can also block reabsorption of bile acids [121]. A few clinical studies indicate an improvement in men’s fertility in terms of sperm count and mobility when royal jelly is administered, due to its naturally increasing testosterone levels in the body [122]. Moreover, its antioxidants reduce the oxidative stress level of liver cells, preventing fat accumulation [123]. Another advantage of using royal jelly is the reduction in tumor cell growth [124]. According to some clinical studies, it can emit antitumoral effects against lung and colorectal cancer due to its bioactive components, which can inhibit cytokines and tumor necrosis factor [125]. Royal jelly represents a great dietary supplement, especially for elders, preventing the appearance of dementia or Alzheimer’s disease [126]. It also promotes bone health and maintains muscle functions. Its flavonoids improve overall immune system functioning [86]. In the coming years, global demand for royal jelly—for consumption, domestic use, and cosmetic use—is projected to increase significantly. This predicted market growth necessitates progressive and permanent development in advanced production techniques and a higher degree of specialization among beekeepers.

## 4. Interaction and Synergistic Potential of CS and Royal Jelly

### 4.1. Structural and Functional Synergy in Nanocomposite Materials

**Figure 6:**
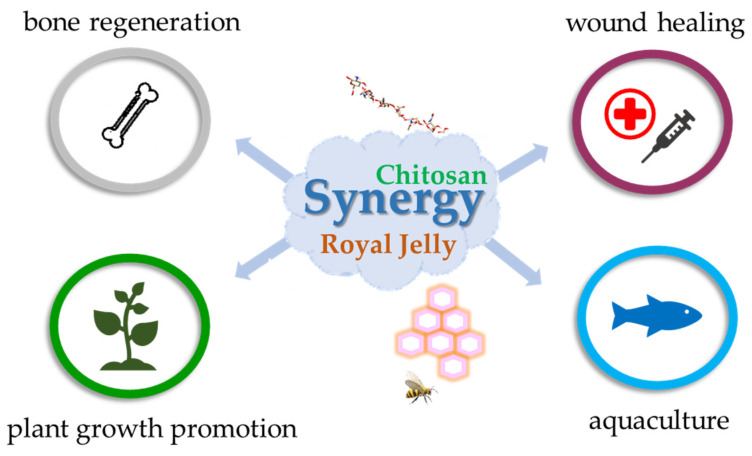
Combined effects of CS and royal jelly across applications such as wound healing, bone regeneration, plant growth, and aquaculture.

In recent years, the scientific literature highlighting extensive research into the synergistic effects of CS and royal jelly has significantly increased. These studies investigate how the combined properties of both materials can create a more effective therapeutic pathway. Individually, CS is a versatile polymer, as outlined in the dedicated section, making it an excellent biomedical candidate, while royal jelly provides powerful antioxidant, anti-inflammatory, and antimicrobial benefits due to its composition, which is rich in bioactive compounds. The combination is synergistic because CS enhances the stability and controlled release of royal jelly’s active components, thereby improving therapeutic outcomes for skin disorders and wound healing processes. This synergy manifests as notable enhancements in mechanical integrity, enhanced antimicrobial efficacy, and accelerated tissue regeneration. The combination’s effectiveness is rooted in royal jelly’s complex biochemical composition, which not only structurally reinforces the CS network, but also provides a potent source of bioactive molecules that complement CS’s intrinsic properties. This section synthesizes the latest diverse findings, from wound and bone healing to advanced nanocomposites and agriculture, demonstrating a broad and feasible principle for developing bio-enhanced materials (Figure 6). The synergistic phenomenon has been extensively investigated to characterize its major therapeutic benefits [127].

For instance, the addition of 5 wt.% royal jelly to a CS matrix results in a nanocomposite with superior mechanical properties, thereby enhancing its biological performance and making it an ideal candidate for advanced wound dressings. This reinforcing effect is quantifiable, demonstrated by an increase in the elastic modulus from 32 MPa to 62 MPa, and in tensile strength from 1.4 MPa to 3.9 MPa [127]. Crucially, the gelatinous, sticky, and amorphous nature of royal jelly allows it to establish covalent bonds and form crosslinks throughout the polymeric network. This stabilization process, often facilitated by freeze-drying, results in a robust and spongy multi-layered microstructure with highly desirable porosity. A notable increase in porosity was also observed, rising from 76% in pure CS to 85% in the composite material containing royal jelly [127]. In conclusion, royal jelly acts not simply as a passive filler but as a dynamic, multifunctional agent that simultaneously chemically and structurally reinforces the CS network.

### 4.2. Antimicrobial and Cytocompatibility Enhancements

CS-based nanoparticles loaded with royal jelly were successfully prepared via ionotropic gelation method [26]. The resulting nanomaterials were characterized by an amorphous and stable structure with a size of less than 500 nm. Functionally, these enhanced nanoparticles demonstrated valuable antibacterial properties, exhibited a favorable in vitro slow-release profile, and showed good response during digestion processes [26]. This fact confirms that embedding royal jelly in a CS matrix is an excellent way to protect it and greatly improves its overall effectiveness and its powerful antimicrobial properties. This study validates the synergistic effect of both CS and Royal jelly.

As detailed in another parallel study, a multi-component film was characterized, which contained a blend of sodium alginate and CS, loaded with royal jelly and other bee derivatives and fortified with green-synthesized silver nanoparticles. This unique formulation produced an inhibition zone diameter of up to 10 mm against bacterial and fungal strains [128]. The proposed mixture formulation exhibited the highest efficacy against Gram-positive bacteria (*Enterococcus faecalis)*, suggesting a synergistic interaction among the components and also against Gram-negative bacteria (*E. coli* and *P. aeruginosa*) [128]. The multi-component film showed the largest inhibition zone against *P. aeruginosa*, indicating a potent synergistic effect when the bee products and silver nanoparticles are combined within the polymeric matrix. This antimicrobial synergy is further amplified at the nanoscale [128]. Moreover, the study demonstrated a desirable, selective outcome: the material reduced cell viability (as measured by an MTT assay), while simultaneously maintaining cellular membrane integrity (confirmed by an LDH assay) [128]. However, the researchers proposed a more complex explanation [128]: the high concentration of monosaccharides derived from honey and royal jelly may modulate the cells’ metabolic pathways. Since the MTT assay measures metabolic activity by tracking the reduction of tetrazolium salt by mitochondrial enzymes, the results may represent a false-positive reduction in viability due to down-regulation of the aerobic glucose oxidation pathway. In sharp contrast, the LDH assay, which directly measures cell membrane damage, showed no significant toxicity [128]. Thus, this discrepancy highlights the importance of employing a multi-assay approach to fully characterize the biological effects of CS and bee derivatives and underscores that the powerful metabolic influence of royal jelly must be carefully considered when optimizing material compositions for therapeutic applications.

### 4.3. Bone Regeneration

CS scaffolds combined with royal jelly achieved superior healing outcomes compared to untreated, CS-only, and even autograft groups [28]. Histopathological analysis showed that the loaded scaffold exhibited superior biodegradability. While CS-only scaffolds remained largely intact after 56 days, loaded scaffolds were completely degraded and replaced by new, regenerated tissue [28]. This highlights royal jelly’s important function: accelerating the biological degradation of the CS scaffold, transforming it from a static placeholder into a dynamic template. Biomechanical tests showed that the healed bones in the CS–royal jelly group were significantly superior to those in the autograft group in terms of yield load and bending stiffness. The overall accelerated, guided regeneration facilitated by royal jelly results in faster and stronger repairs [28]. The CS–bee derivative group showed significantly higher radiographic scores at 56 days post-surgery than the untreated and CS-only groups, performing statistically on par with the gold-standard autograft group. Royal jelly’s rich biochemical composition is the primary driver of this synergy, as its components directly stimulate essential cellular processes. For instance, Apisin, a major glycoprotein, is shown to increase the proliferation of neonatal skin fibroblasts and promote the differentiation of MC3T3-E1 cells into osteoblasts. This molecular-level action directly explains the enhanced tissue filling and accelerated bone regeneration observed in the *in vivo* rat study. Additionally, the fatty acid 10-hydroxy-2-decenoic acid (10-HDA), a key quality marker for royal jelly, is very well-known for its antibiotic activity against various pathogens. This compound likely contributes to the potent antimicrobial efficacy of the composite material, protecting it from infection [28].

Moreover, royal jelly can serve as a natural “mediator of synthesis” for silver nanoparticles [28]. This environmentally friendly approach offers an alternative to traditional methods by mediating the synthesis and subsequent embedding of the nanoparticles directly into a CS matrix. The obtained nanomaterial effectively suppressed bacterial film formation in pathogenic yeasts and prevented conversion to the hyphal form in *C. albicans* [28]. Last but not least, this outcome demonstrates a powerful three-way synergistic relationship: royal jelly enables the synthesis of potent silver nanoparticles, and the CS matrix acts as a stable, biocompatible carrier for their controlled delivery, leading to a highly effective, multi-pronged attack on pathogens.

### 4.4. Broader Applications in Agriculture and Aquaculture

Foliar application of mixed compounds has been shown to improve the growth, yield, and quality of garlic plants in agriculture. In a two-season study, spraying garlic cultivars with varying concentrations of CS and bee derivative resulted in a considerable increase in plant height and leaf number compared to control samples [129]. This enhancement in vegetative growth was reflected in higher total yield and improved bulb quality. The positive effects of royal jelly are attributed to its rich content of nutrients that function as potent bio-stimulants and promote plant growth. CS, in turn, enhances nutrient uptake, elicits defense mechanisms, and, as an edible coating, reduces post-harvest weight loss and deterioration of bulbs during storage [129]. The fact that this combination is effective across both mammalian and plant systems suggests a fundamental, universal principle of biological enhancement, where this bee derivative provides a rich source of biological nutrients, and CS provides a reliable, biodegradable delivery system and protective layer [129]. A parallel study in aquaculture provides further evidence of this synergy, specifically with another bee product, bee venom, encapsulated in CS nanoparticles [130]. A study on Pacific white shrimp showed that a diet supplemented with loaded nanoparticles significantly enhanced shrimp growth, immunity, and resistance to a *Vibrio parahaemolyticus* challenge [130]. This demonstrates a clear precedent even for the potential of loaded nanoparticles with royal jelly in aquaculture. Finally, the ability of CS nanoparticles to act as an effective carrier for natural bioactive compounds, as shown with bee venom, strongly supports the hypothesis that a similar delivery system with royal jelly could yield comparable benefits in enhancing the health and productivity of aquatic species [130].

## 5. Key Conclusions and Outlook for Future Research

This comprehensive review confirms that the synergy between CS and royal jelly leads to the formation of a potent and versatile platform with applications ranging from advanced wound dressings and bone tissue regeneration to agricultural bio-stimulants and aquaculture health. Its therapeutic efficacy, observed in diverse studies, is rooted in a true synergistic relationship, where the unique properties of each component are mutually enhanced. The combined effects of CS and royal jelly create a highly promising bio-enhanced composite material. Significantly, the CS structure provides a pH-responsive, controlled release mechanism which controls and protects royal jelly’s fragile compounds and enables targeted delivery, thereby minimizing systemic side effects. The primary concern would be long-term stability. Royal jelly’s components, particularly proteins and fatty acids, are sensitive to degradation from light, heat, and moisture. Future work must focus on developing standardized encapsulation techniques which preserve bioactivity upon integration into scaffolds, hydrogels, or nanoparticles, ensuring a reliable shelf life for topical and injectable formulations. Currently, the precise molecular mechanism of synergy still remains largely unknown. While the platform provides structural integrity and antimicrobial action, the specific components responsible for enhanced anti-inflammatory and regenerative effects must be definitively identified. More research is needed using advanced analytical techniques to map the specific intermolecular binding sites between the active components. Elucidating this mechanism represents the key to rationally designing materials with maximized therapeutic efficacy. Furthermore, comprehensive studies are necessary to fully elucidate the precise pathways and long-term biological impacts of these combined effects, particularly through more sophisticated in vitro models. The capacity of royal jelly to mediate nanoparticle synthesis presents an exciting avenue for developing new targeted therapeutic delivery systems. Ultimately, the convergence of natural biopolymers and nanotechnology, exemplified by the CS–royal jelly platform, holds immense promise for developing the next generation of sustainable, highly effective functional materials for diverse biomedical applications.

## Figures and Tables

**Table 1:** CS-based materials in wound healing therapy.

Formulations	Applications	References
Lutein-loaded carboxymethyl CS hydrogels	Acute/chronic wounds and burns	[53,54]
Allantoin-loaded CS nanoparticles	Gastric ulcers	[55]
Xylan–CS-based films	Antibacterial activity against MRSA strain	[56]
Sodium hyaluronate/CS foams	Hemostasis	[57]
CS nanofibers	Antibacterial activity and alleviating inflammatory responses	[58]
CS-based sponges	Preventing infection	[59]
CS bandages	Antiseptic, topical skin infections	[60]
CS porous membranes	Analgesic/burns	[61]
CS/glycerol micropatterned composite	Hemostasis and inflammations	[62]
CS-based-photo-crosslinked hydrogel	Treating infections	[63]
Phosphorylated CS	Dermal healing	[64]
CS patches	Antimicrobial activity, hemostasis	[65]

## Data Availability

No new data were created or analyzed in this study. Data sharing is not applicable to this article.
